# Oral Immunization of Recombinant *Lactococcus lactis* and *Enterococcus faecalis* Expressing Dendritic Cell Targeting Peptide and Hexon Protein of Fowl Adenovirus 4 Induces Protective Immunity Against Homologous Infection

**DOI:** 10.3389/fvets.2021.632218

**Published:** 2021-02-23

**Authors:** Zhipeng Jia, Chunli Ma, Xuelian Yang, Xinghui Pan, Guangxing Li, Dexing Ma

**Affiliations:** ^1^Heilongjiang Key Laboratory for Laboratory Animals and Comparative Medicine, College of Veterinary Medicine, Northeast Agricultural University, Harbin, China; ^2^Heilongjiang Key Laboratory for Laboratory Animals and Comparative Medicine, Harbin, China; ^3^Food College, Northeast Agricultural University, Harbin, China

**Keywords:** FAdV-4, Hexon, *Lactococcus lactis*, *Enterococcus faecalis*, dendritic cell-targeting, oral immunization

## Abstract

Hepatitis-hydropericardium syndrome (HPS) causes severe economic losses in the global poultry industry. The present study aims to explore oral immunization of recombinant *Lactococcus lactis* and *Enterococcus faecalis* expressing Hexon protein of fowl adenovirus 4 (FAdV-4). The bacteria *L. lactis* NZ9000 and *E. faecalis* MDXEF-1 were, respectively, modified as host strain to deliver truncated Hexon protein (ΔHexon) or ΔHexon protein fusing with dendritic cell (DC) targeting peptide (DC-ΔHexon) on the surface of bacteria. The expression of target protein in *L. lactis* NZ9000 and *E. faecalis* MDXEF-1 were detected by western blot. To evaluate the immune responses and protective efficacies provided by the live recombinant bacteria, chickens were immunized with the constructed ΔHexon-expressing bacteria three times at 2-week intervals, then experimentally challenged with hypervirulent FAdV-4/GX01. The results showed that oral immunizations with the four ΔHexon-expressing bacteria (NZ9000/ΔHexon-CWA, NZ9000/DC-ΔHexon-CWA, MDXEF-1/ΔHexon-CWA, and MDXEF-1/DC-ΔHexon-CWA), especially the two bacteria carrying DC-targeting peptide, stimulated higher levels of ΔHexon-specific sera IgG and secretory IgA (sIgA) in jejunal lavage fluid, higher proliferation of peripheral blood lymphocytes (PBLs) and higher levels of Th1/Th2-type cytokines, along with significantly decreased virus loads in liver and more offered protective efficacies against FAdV infection compared with PBS and empty vector control groups (*p* < 0.01). For chickens in the group MDXEF-1/DC-ΔHexon-CWA, the levels of aspartate transaminase (AST), alanine transaminase (ALT) and lactate dehydrogenase (LDH) in sera, and the virus loads in livers were significantly decreased vs. the other three ΔHexon-expressing bacteria (*p* < 0.01). The pathological changes in the hearts, livers, spleens and kidneys of chickens in MDXEF-1/DC-ΔHexon-CWA group were relatively slight compared to infection control group and other three ΔHexon-expressing bacteria groups. The rate of protection in MDXEF-1/DC-ΔHexon-CWA group was 90%. The present work demonstrated that cell surface-displayed target protein and immune enhancers in *L. lactis* and *E. faecalis* might be a promising approach to enhance immunity and immune efficacy against pathogen FAdV-4 infection.

## Introduction

Fowl adenoviruses (FAdVs) are non-enveloped, double-stranded DNA viruses belonging to the Aviadenovirus genus of the Adenoviridae family. FAdVs are currently divided into 5 species (A–E) and 12 serotypes (1–7, 8a, 8b, 9–11) (1, 2). FAdV-4 usually infects 3–6 weeks old broilers showing a mortality rate of up to 80%, and causes accumulation of transparent or straw-colored fluid in the pericardial sac and hepatitis (3–5). Hepatitis-hydropericardium syndrome (HPS) is one of the typical gross pathological changes in FAdV-4 infected chickens, which has been widely reported in several countries and regions such as Middle East (6), Germany (7), Korea (8), Malaysia (9), and Japan, India and Pakistan (10). In China, HPS caused by novel hypervirulent FAdV-4 has been reported since 2015 (11), which leads to significant economic losses to the broiler poultry industry. Therefore, exploration of vaccine aiming at preventing HPS has become a research hot spot.

Currently, inactivated vaccines and live attenuated vaccines prepared based on the FAdV-4 ON1 strain were characterized to be immunogenic and effective against FAdV-4 infection (12). Considering the potential reversion to virulence for attenuated vaccines, and the potential tumorigenicity of adenoviruses, the novel genetic engineering vaccines are still necessary to be explored. It is generally accepted that mucosal immunity represents the first line of defense, and mucosal vaccination can evoke protective mucosal immune responses against highly contagious virus *via* the oral route (13). In recent years, lactic acid bacteria (LAB) has been widely used as vehicles to deliver important antigens of pathogens, such as the circumsporozoite protein of *Plasmodium falciparum* (14), spike protein of SARS-CoV-2 (15), the heavy-chain antigen of *Clostridium botulinum* serotype A neurotoxin and the *Bacillus anthracis* protective antigen (16). Previous studies have shown that the expression of FAdV-4 structural proteins, including Hexon, Penton, Fiber 1 and Fiber 2, in *Escherichia coli* and other expression systems could be used to develop subunit vaccines (17–22). However, the protective efficiencies of live recombinant LAB delivering structural proteins of FAdV-4 against homologous challenge have not been assessed until now. We and others also reported that live recombinant *Lactococcus lactis* NZ9000 delivering *Eimeria* 3-1E protein (23) and avian hepatitis E virus (aHEV) ORF2 protein (24), *Enterococcus faecalis* displaying *Eimeria* 3-1E protein (25), *Lactobacillus plantarum* expressing *Eimeria tenella* MIC2 protein (26) to some extent provided protective efficacies against poultry disease. Moreover, previous studies have confirmed that oral vaccination with genetically modified bacteria delivering dentritic cell (DC) targeting peptides and immunogenic antigens of pathogens enhanced antigen-specific mucosal immunity against homologous pathogen infection (27–30).

Based on the previous studies, we speculated that the effective mucosal and humoral immune responses evoked by oral immunization would be a promising means to prevent HPS caused by FAdV. In the present study, *L. lactis* NZ9000 and *E. faecalis* MDXEF-1 were used as host strains to express ΔHexon proteins of FAdV, respectively. Meanwhile, DC targeting peptide was introduced to fuse with anchored ΔHexon proteins to enhance the antigenic-specific immune responses. Then, the recombinant *L. lactis* and *E. faecalis* were used to immunize chickens *via* oral route, and the immune responses and protection against FAdV-4 challenge were evaluated.

## Materials and Methods

### Bacterial Strains, Plasmids, and Virus

Details of the strains and plasmids used in this experiment are listed in Table 1. *Lactococcus lactis* NZ9000, *E. faecalis* MDXEF-1 (25), and derivative strains were grown at 30°C in GM17 broth (M17 containing 0.5% glucose, Luqiao, Beijing) without shaking or on GM17 culture plate with 1.5% agar. *E. coli* strains were cultured on Luria-Bertani (LB) medium (Hopebiol) at 37°C. Chicken hepatoma cell line (LMH) (ATCC) was cultured in DMEM medium supplemented with 10% fetal bovine sera at 37°C in 5% CO_2_. FAdV-4 strain GX01 (FAdV-4/GX01, GenBank no. MH229946.1) used in the present study was isolated from a natural case of HPS by Prof. Guangxing Li in our laboratory. The virus was purified and propagated in the LMH cells.

**Table 1:** Strains and plasmids used in this study.

**Bacterial strain or plasmids**	**Relevant characteristics**	**Source or references**
**Strain**		
*E. coli* DH5α	SupE44ΔlacU169(φ80 lacZΔM15) hsdR17 recA1 endA1 gyrA96 thi-1 relA1, plasmid-free.	TaKaRa, Dalian, China.
*E. coli* BL21(DE3)	F-ompT hsdSB (rB- mB-) gal dcm (DE3), plasmid-free.	TaKaRa, Dalian, China.
*L. lactis* subsp. cremoris NZ9000	Derivate strain of MG1363, with nisR and nisK genes for nisin induction, plasmid-free strain.	NIZO
*E. faecalis* MDXEF-1	Isolated from chicken and reserved by our laboratory (Chinese patent ZL201410817717.5)	(25)
*E. coli* DH5α/pMD19T-ΔHexon	With plasmid pMD19T-ΔHexon in *E. coli* DH5α.	This study
*E. coli* BL21(DE3)/pET30a-ΔHexon	With plasmid pET30a-ΔHexon in *E. coli* BL21(DE3).	This study
NZ9000/pTX8048	With plasmid pTX8048 in *L. lactis* cremoris NZ9000.	This study
MDXEF-1/pTX8048	With plasmid pTX8048 in *E. faecalis* MDXEF-1.	This study
NZ9000/pTX8048-SP-ΔHexon-CWA	With plasmid pTX8048-SP-ΔHexon-CWA in *L. lactis* cremoris NZ9000.	This study
NZ9000/pTX8048-SP-DC-ΔHexon-CWA	With plasmid pTX8048-SP-DC-ΔHexon-CWA in *L. lactis* cremoris NZ9000.	This study
MDXEF-1/ΔHexon-CWA	With plasmid pTX8048-SP-ΔHexon-CWA in *E. faecalis* MDXEF-1.	This study
MDXEF-1/pTX8048-SP-DC-ΔHexon-CWA	With plasmid pTX8048-SP-DC-ΔHexon-CWA in *E. faecalis* MDXEF-1.	This study
**Plasmid**		
pMD19T	High-efficiency TA cloning vector.	TaKaRa, Dalian, China
pET30a	Escherichia coli expression vector.	Novagen, Madison, WI
pMD19T-ΔHexon	With fragment encoding ΔHexon protein in pMD19T.	This study
pET30a-ΔHexon	With fragment encoding ΔHexon protein in pET30a.	This study
pTX8048	With fragment encoding signal peptide of secretion protein Usp45 (SP).	(31)
pTX8048-SP-ΔORF2-CWA	With fragment encoding signal peptide of secretion protein Usp45 (SP) and ΔORF2 protein in anchored form, no dendritic cell targeting peptides.	(24)
pTX8048-SP-DC-3-1E-CWA	With fragment encoding signal peptide of secretion protein Usp45 (SP) and 3-1E protein in anchored form, contains dendritic cell targeting peptides.	(25)
pTX8048-SP-ΔHexon-CWA	With fragment encoding signal peptide of secretion protein Usp45 (SP) and ΔHexon protein in anchored form, no dendritic cell targeting peptides.	This study
pTX8048-SP-DC-ΔHexon-CWA	With fragment encoding signal peptide of secretion protein Usp45 (SP) and ΔHexon protein in anchored form, contains dendritic cell targeting peptides.	This study

### Animals

Two male New Zealand rabbits weighing about 2 kg were obtained from a rabbit farm. Specific-pathogen-free (SPF) White Leghorn chickens and embryos were purchased from the Harbin Veterinary Research Institute (Heilongjiang, China). 9-day-old SPF chicken embryos were infected with serial 10-fold dilutions of FAdV-4/GX01 (0.2 mL) onto each embryo's chorioallantoic membranes and incubated at 37°C for 10 days to calculate the 50% embryo lethal dose (ELD_50_) according to the formula of Reed and Muench (32). In this study, animals were maintained on a 12-h light/12-h dark cycle, with unrestricted access to food and drinking water. Animal experiments were performed according to the regulations (SRM-12) of the Ethical Committee for animal sciences in Northeast Agricultural University, Heilongjiang Province, PR China.

### Preparation of Polyclonal Antisera Against ΔHexon

Virus DNA was extracted from semi-purified viral suspension using TaKaRa MiniBEST Viral RNA/DNA Extraction Kit Ver.5.0 (Takara, Beijing, China) according to the manufacturer's instructions. The target ΔHexon gene fragments were amplified using primers pair ΔHexon-F2 and ΔHexon-R2 (Table 2) using prepared DNA as a template. The amplification parameters were as follows: 95°C for 5 min, followed by 35 cycles of 95°C for 30 s, 57°C for 30 s, and 72°C for 75 s, 72°C for 10 min and a 4°C hold. The amplified ΔHexon gene fragments were inserted into *Bam*H I and *Xho* I sites of pET30a vectors (Novagen, Madison, WI) to generate plasmid pET30a-ΔHexon, which were then transformed into *E. coli* BL21(DE3) competent cells to produce recombinant positive *E. coli* cells. The positive bacteria were induced with the final concentration of 1 mM Isopropyl β-D-Thiogalactopyranoside (IPTG) (Solarbio, Beijing, China) for 7 h at 37°C when optical density at 600 nm (OD_600_) reached 0.5 or 0.6. The harvested cells were lysed by sonication, and the expression of ΔHexon protein was analyzed by sodium dodecyl sulfate-polyacrylamide gel electrophoresis (SDS-PAGE). The expression and purification of ΔHexon protein were carried out as previously described (35). Briefly, the target ΔHexon protein fused with a 6× His tag was purified using a His-tag Protein Purification Kit (Beyotine Biotechnology) according to the manufacturer's protocol. 2 mg of purified ΔHexon protein mixed with 2 ml of complete Freund's adjuvant (Sigma, USA) was used to immunize New Zealand white rabbits. The second, third, and fourth immunizations were boosted at 2-weeks intervals as the primary immunization except that the complete Freund's adjuvant was replaced by incomplete Freund's adjuvant (Sigma, USA). The specificity of antisera was detected by western blot, as previously described (35). The titer of the antisera was determined by enzyme linked immunosorbent assay (ELISA). In brief, 100 μL of purified ΔHexon protein (10 μg/mL) was added to each well in a 96-well plate and incubated overnight at 4°C. The plate was washed three times with PBST (PBS containing 0.05% Tween 20), then blocked with 5% skimmed milk at 37°C for 1.5 h. 100 μL of two-fold serially diluted sera was added to each well and incubated for 1.5 h at 37°C. After washing, the plates were incubated with HRP-conjugated goat anti-rabbit IgG (Sigma-Aldrich) diluted at 1:5000 for 1 h at 37°C. 1 mg/mL of o-phenylenediamine and 0.01% H_2_O_2_ were added (100 μL per well), and the reaction was stopped by 2 M H_2_SO_4_. The absorbance was measured at 490 nm using a reader (Bio-Rad, USA).

**Table 2:** Primer sequences with their corresponding PCR product size.

**Name of primer**	**Primer sequences (5^**′**^-3^**′**^)**	**Enzyme**	**PCR Product (base pairs)**	**Source or references**
ΔHexon -F2	CGCGGATCCGCGACTCCGCGGCTCCAGTAT	*Bam*H I	1,230 bp	This study
ΔHexon -R2	GAGACTCGAGAGTGCCGAAGTAGAAGTTGG	*Xho* I		
ΔHexon -F3	CGCGGATCCGCGACTCCGCGGCTCCAGTAT	*Bam*H I	1,230 bp	This study
ΔHexon -R3	GGGGTACCAGTGCCGAAGTAGAAGTTGG	*Kpn*I		
52K-F	ATGGCGCAGATGGCTAAGG	/	176 bp	(33)
52K-R	AGCGCCTGGGTCAAACCGA	/		
Ch IL-2-F	GTGGCTAACTAATCTGCTGTCC	/	105 bp	(24)
Ch IL-2-R	GTAGGGCTTACAGAAAGGATCAA	/		
Ch IL-4-F	CTGTGCCCACGCTGTGCTTA	/	83 bp	This study
ChIL-4-R	GGAAACCTCTCCCTGGATGTCA	/		
ChIL-10-F	GGCTCACTTCCTCCTCC	/	112 bp	This study
ChIL-10-R	TGACTTTCACCTGCAGATG	/		
ChIFN-γ-F	CAAAGCCGCACATCAAACA	/	80 bp	(24)
ChIFN-γ-F	TTTCACCTTCTTCACGCCATC	/		
Chβ-actin-F	GCCAACAGAGAGAAGATGACAC	/	138 bp	(34)
Chβ-actin-R	GTAACACCATCACCAGAGTCCA	/		

### Identification of Recombinant *L. lactis* and *E. faecalis* Expressing ΔHexon Protein

**Figure 1:**
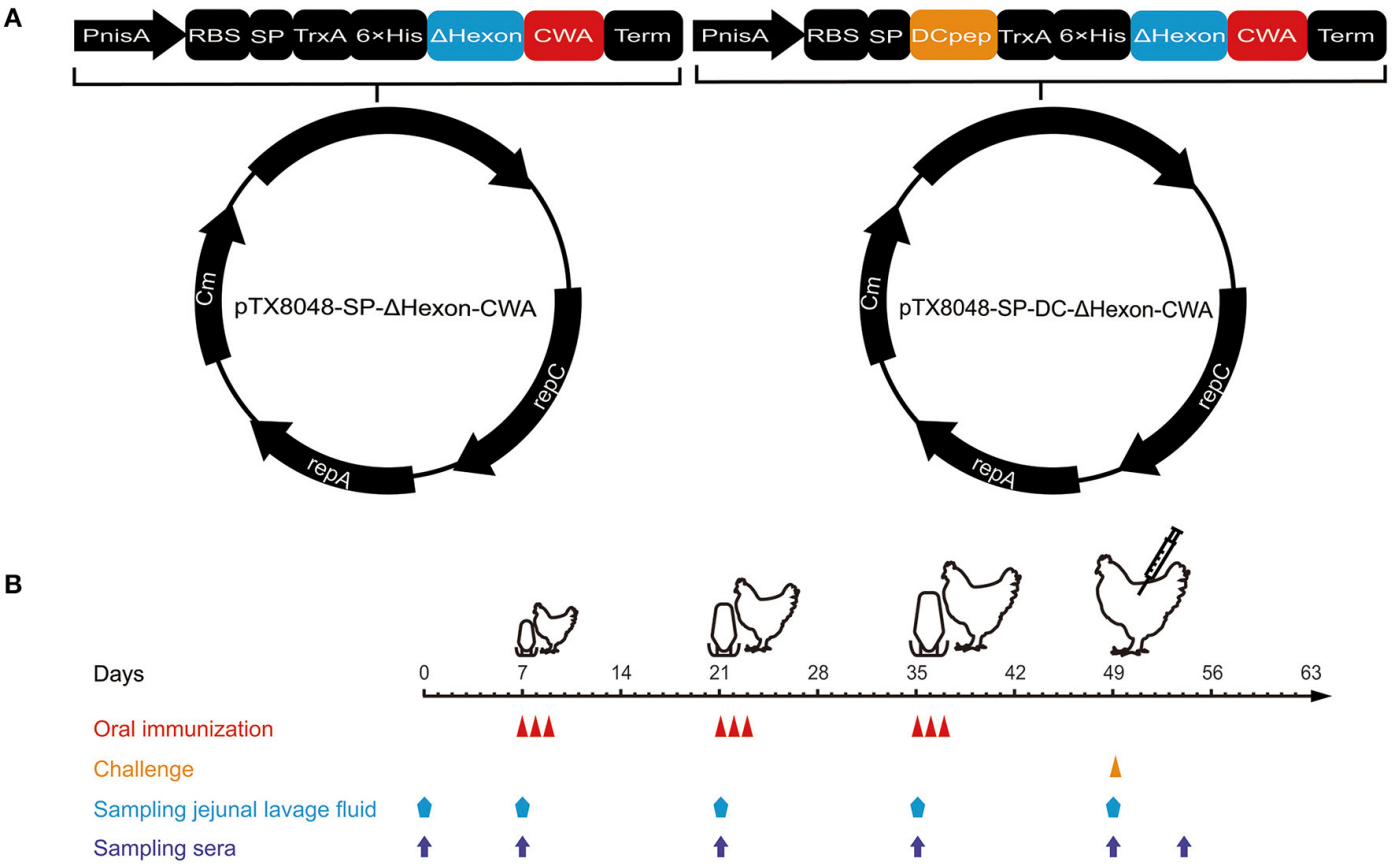
Procedures of immunization, viral challenge, and sampling. **(A)** Schematic diagram of the recombinant plasmids pTX8048-SP-ΔHexon-CWA and pTX8048-SP-DC-ΔHexon-CWA. **(B)** The immunization procedures and sampling schedules. Chickens were immunized with the constructed ΔHexon-expressing bacteria three times at 2-week intervals and experimentally challenged with hypervirulent FAdV-4/GX01 strain on day 14 post the third immunization.

The ΔHexon gene fragment was amplified by primers pair ΔHexon-F2 and ΔHexon-R3 (Table 2), and subcloned into the *Bam*H I and *Kpn* I sites of pTX8048-SP-CWA (containing cell wall-anchored sequence) or pTX8048-SP-DC-CWA (containing cell wall-anchored sequence and dendritic cell-targeting peptide) to generate plasmids pTX8048-SP-ΔHexon-CWA and pTX8048-SP-DC-ΔHexon-CWA, respectively (Figure 1A). The above two positive plasmids were confirmed by nucleotide sequence analysis, then were transformed into *L. lactis* NZ9000 and *E. faecalis* MDXEF-1 competent cells by electroporation, respectively. The characterized positive bacteria were named NZ9000/ΔHexon-CWA, NZ9000/DC-ΔHexon-CWA, MDXEF-1/ΔHexon-CWA, and MDXEF-1/DC-ΔHexon-CWA, respectively. Meanwhile, recombinant bacteria NZ9000/pTX8048 and MDXEF-1/pTX8048 were used as control. Recombinant positive *L. lactis* and *E. faecalis* were cultured to OD_600_ values of 0.5, then induced by a final concentration of 5 ng/mL nisin (Sigma-Aldrich) for 4 h. The cell wall-anchored protein samples were prepared as previously described (31). In brief, cell pellets were washed and resuspended in TES (10 mM Tris-HCl pH 8.0, 1 mM EDTA, 25% sucrose). The buffer TES-LMR (TES containing 1 mg/ml lysozyme, 0.1 mg/ml mutanolysin, 0.1 mg/ml RNase) was applied to digest cell walls. After centrifugation, the cell walls were pelleted and removed, and the cell wall-anchored proteins in the supernatant were precipitated with final concentration of 16% trichloroacetic acid (TCA). The target cell wall-anchored protein was resuspended. The prepared protein samples were separated by SDS-PAGE, and electrophoretically transferred to nitrocellulose membranes. The membranes were incubated with rabbit anti-ΔHexon polyclonal antisera (1:1000), then reacted with horseradish peroxidase (HRP)-conjugated goat anti-rabbit IgG antibody (1:2000) (Sigma, USA). The immunoreactive protein bands were visualized using an ECL chemiluminescence detection kit (BeyoECL Moon).

To further testify the target ΔHexon protein was displayed on the surface of recombinant *L. lactis* NZ9000 and *E. faecalis* MDXEF-1, indirect immunofluorescence assay (IFA) was performed. Briefly, the recombinant positive *L. lactis* and *E. faecalis* were cultured to OD_600_ values of 0.5, and then induced by a final concentration of 5 ng/mL nisin (Sigma-Aldrich) for 4 h. The bacterial cultures were washed twice with sterile PBS (pH7.2), followed by centrifugation at 10,000 × g for 10 min. Then the pellets were incubated with rabbit polyclonal antisera against ΔHexon (1:200) (primary antibody) and goat anti-rabbit IgG conjugated to fluorescein isothiocyanate (FITC) (1:50, Solarbio) (secondary antibody). After washing, the fluorescence on the surface of recombinant bacteria were observed using fluorescence microscope (Leica DM2000).

### Oral Immunization and Challenge Experiment

Animal grouping, immunizations, and challenges were displayed in Supplementary Table 1. A total of three immunizations were performed at 2-weeks intervals (Figure 1B). Tissue samples of liver, spleen, and heart were collected, and sera and jejunal lavage fluid were prepared from five chickens randomly selected in each group at each time point (Figure 1B). The jejunum tissue (10 cm in length) from chicken in each group was flushed twice with a total of 5 ml of cold PBS (pH 7.2) containg final concentration of 1 mM PMSF (Solarbio). Then the collected jejunal lavage fluid was centrifuged at 800 × g for 10 min at 4°C, and the supernatants were harvested and stored at −80°C until assays. On day 14 after the third immunizations, all chickens except those in PBS control group were challenged with FAdV-4/GX01 (200 μL, containing 10^5.2^ ELD_50_) (Supplementary Table 1).

### Humoral Immune Responses

The levels of specific IgG in sera and sIgA in jejunal lavage fluid were detected by indirect ELISA as previously described (24). Briefly, ΔHexon protein (1 mg/mL) was coated on the 96-well plate (100 μL per well). The prepared sera diluted at 1:50 and jejunal lavage fluid diluted at 1:10 were added, respectively, and incubated at 37°C for 1 h. HRP-conjugated goat anti-chicken IgG or goat anti-chicken IgA (Abcam, USA) were used as secondary antibodies, respectively. 100 μL of o-phenylenediamine (1 mg/mL) and 0.01% H_2_O_2_ were added to each well, and the reaction was stopped by 2 M H_2_SO_4_. The absorbance was measured at 490 nm using a reader (Bio-Rad, USA). Each sample was tested in triplicate.

### Cytokine Levels in Spleen

On day 14 after the third immunization, the levels of chicken IL-2 (ChIL-2), chicken interferon-gamma (ChIFN-γ), chicken IL-4 (ChIL-4), and chicken IL-10 (ChIL-10) in spleens of all euthanized chickens were detected by quantitative real-time PCR (qPCR). Briefly, total RNA was extracted from spleen using Trizol reagent (Invitrogen, Carlsbad, CA). cDNA was synthesized from 1 μg of total RNA using Prime Script RT reagent Kit (TaKaRa Biotech Corp., Dalian, China) according to manufacturer's instructions. qPCR was carried out using SYBR® Premix Ex Taq^TM^ II (Tli RNase H Plus) (TaKaRa Biotech Corp., Dalian, China). qPCR was performed following the minimum information for publication of quantitative real-time PCR experiments (MIQE) guidelines (36). β-actin was used as a reference gene for normalization. Primers pairs used in the present study are listed in Table 2. For each 100-fold diluted cDNA sample, amplification efficiencies of all target genes and the reference gene were similar, and the 2^−ΔΔCt^ method was used to analyze the relative quantification of the target gene (37).

### Lymphocyte Proliferation in Peripheral Blood

To determine the proliferation of peripheral blood lymphocytes (PBLs) on day 14 after the final vaccination, peripheral blood of chickens in each group (*n* = 5) was collected *via* wing vein, and PBLs were isolated using lymphocyte separation medium (1.077 g/mL) (Tianjin Haoyang Biological Manufacture, China). The isolated cells were modulated to a final concentration of 5 × 10^6^ cells/mL. The proliferation of PBLs were determined with the Cell Counting Kit-8 solution (CCK-8, Bimake). Briefly, cell suspension containing RPMI 1640 medium supplemented with 10% fetal bovine sera was added into a 96-well plate (100 μL per well) with eight duplicates and incubated at 37°C for 24 h in a 5% CO_2_ incubator. Cells were stimulated with 5 μg/mL of recombinant ΔHexon protein for 48 h, then 10 μL of Cell Counting Kit-8 solution (CCK-8, Bimake) was added into each well to incubate at 37°C for another 4 h. The CellTiter 96® AQueous Non-Radioactive Cell Proliferation Assay (Promega, Fitchburg, WI, USA) was evaluated according to the manufacturer's instructions. The value of OD_490nm_ in each well was measured. Each sample was tested in triplicate. Cells stimulated with 5 μg/mL of concanavalin A (ConA, Sigma) or incubated with cell culture medium was used as a positive and negative control, respectively.

### Detection of Hepatic Function and Viral Load in Liver

The levels of aspartate transaminase (AST), alanine transaminase (ALT), albumin (ALB), total protein (TP), and lactate dehydrogenase (LDH) in sera were determined using the corresponding detection kit (Jiancheng Biological Engineering Institute, Nanjing, Jiangsu) according to the manufacturer's protocol. Each sample was detected in triplicate. DNA of FAdV was extracted by TaKaRa MiniBEST Viral RNA/DNA Extraction Kit Ver.5.0 (Takara, Beijing, China), and the virus load in liver was quantified using qRT-PCR as previously described (33). The primer pair 52K-F/52K-R are listed in Table 2.

### Necropsy and Histopathology

All the birds were euthanized and necropsied. Several organs, including hearts, livers, spleens, and kidneys, were collected for recording gross pathological changes on 5 days past infection (dpi). The above-collected samples were immersed in 10% neutral buffered formalin, embedded in paraffin, sectioned, and stained with hematoxylin-eosin (HE). The histopathological changes in the prepared slides were observed using a light microscope (Nikon, EX200).

### Statistical Analysis

SPSS24 (SPSS/IBM, Chicago, IL, USA) and Prism7.0 (GraphPad Software, La Jolla, CA, USA) was used for one-way ANOVA and Duncan's multiple-comparison procedures of the data. All data are expressed as mean ± standard deviation (SD). A *p*-value of < 0.05 is considered to be statistically significant, and a *p*-value of < 0.01 is considered to be highly significant.

## Results

### Production of Polyclonal Antisera Against ΔHexon Protein

**Figure 2:**
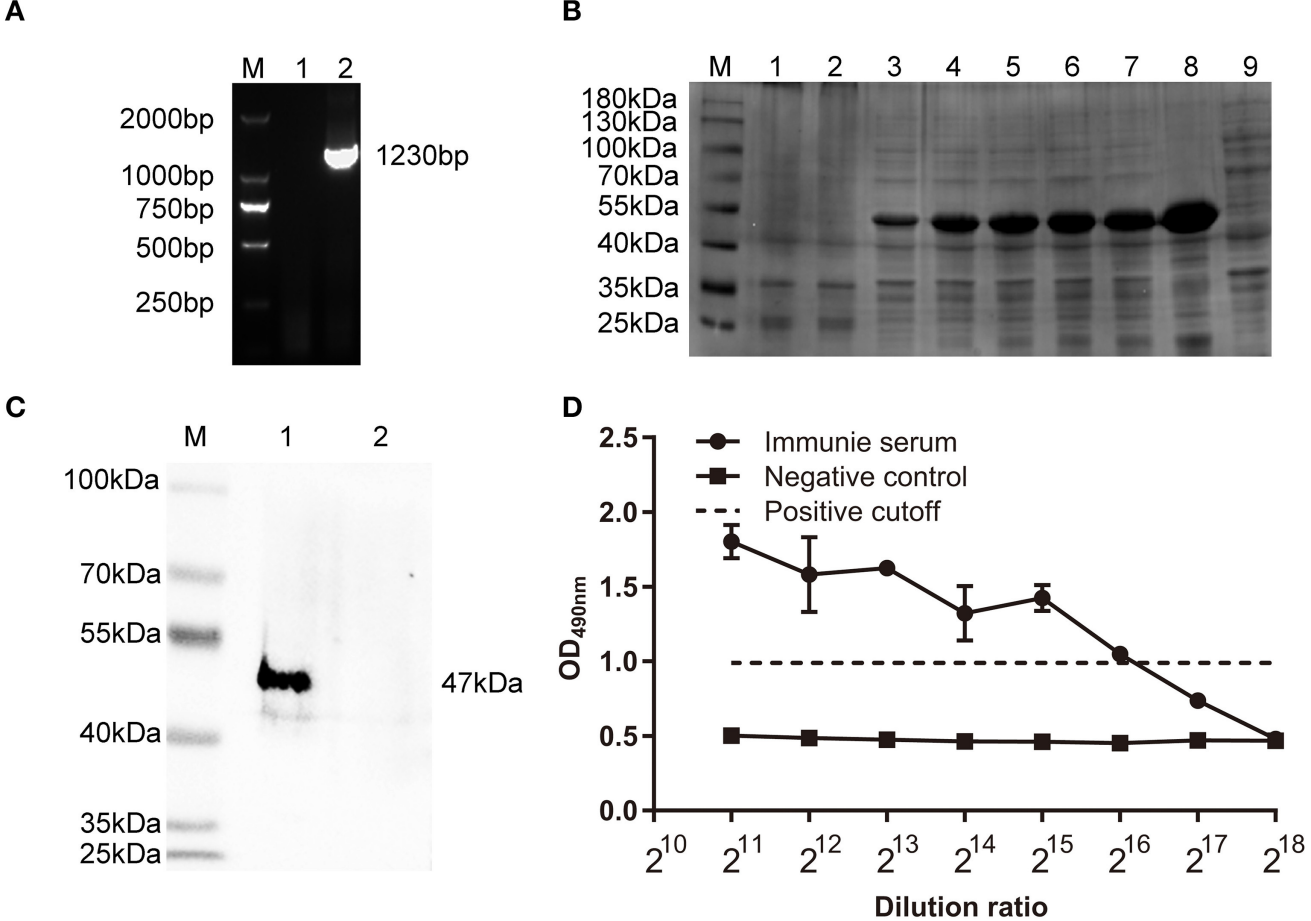
Characterization of polyclonal antisera against ΔHexon protein of FAdV. **(A)** Identification of recombinant plasmid pET30(a)-ΔHexon by PCR. Lane M, DL 2000 DNA Marker; Lane 1, Negative control; Lane 2, Identification of pET30(a)-ΔHexon by PCR. **(B)** SDS-PAGE analysis of expressed ΔHexon protein in *E. coli*. Lane M, protein molecular weight marker (Thermo Scientific™); Lane 1, Proteins in recombinant bacteria *E. coli* BL21/pET30a; Lane 2, Proteins in recombinant bacteria *E. coli* BL21/pET30a-ΔHexon without induction by Isopropyl β-D- Thiogalactopyranoside (IPTG); Lane 3-7, Proteins in recombinant bacterial *E. coli* BL21/pET30a-ΔHexon induced by IPTG for 1, 2, 4, 5, 6 h; Lane 8, Cell pellet of sonicated recombinant bacteria *E. coli* BL21/pET30a-ΔHexon induced by IPTG for 7 h; Lane 9, Cell supernatant of sonicated recombinant bacteria *E. coli* BL21/pET30a-ΔHexon induced by IPTG for 7 h. **(C)** Purified ΔHexon proteins were separated by SDS-PAGE, transferred to nitrocellulose membranes, and probed with prepared rabbit anti-ΔHexon polyclonal sera. The expected bands were observed. Lane M, protein molecular weight marker (Thermo Scientific™). Lane 1, ΔHexon protein from *E. coli* BL21/pET30a-ΔHexon; Lane 2, protein from *E. coli* BL21/pET30a. **(D)** Enzyme linked immunosorbent assay (ELISA) was used to determine titers of rabbit anti-ΔHexon polyclonal antisera. 100 μL of recombinant ΔHexon protein (10 μg/mL) was coated. The prepared sera were diluted in 2-fold series and added into each well. The prepared sera from chickens before immunization was used as the negative control. The cut off value was 2.1 times of the OD_490_ value of the negative control, the titer of prepared rabbit anti-ΔHexon polyclonal sera determined by ELISA was 1:2^16^.

Identification of recombinant plasmid pET30(a)-ΔHexon by PCR showed an expected fragment of 1230 bp (Figure 2A). The FAdV-4 ΔHexon protein (from 10 to 420 aa) was expressed in *E. coli*. BL21, and SDS-PAGE displayed a protein band of ~50 kDa (Figure 2B), which showed positive immunoreaction with rabbit anti-ΔHexon sera in western blot assays (Figure 2C). The titer of the prepared rabbit anti-ΔHexon protein polyclonal antisera was 1:2^16^ (Figure 2D).

### Expression of ΔHexon Protein in *L. lactis* NZ9000 and *E. faecalis*

**Figure 3:**
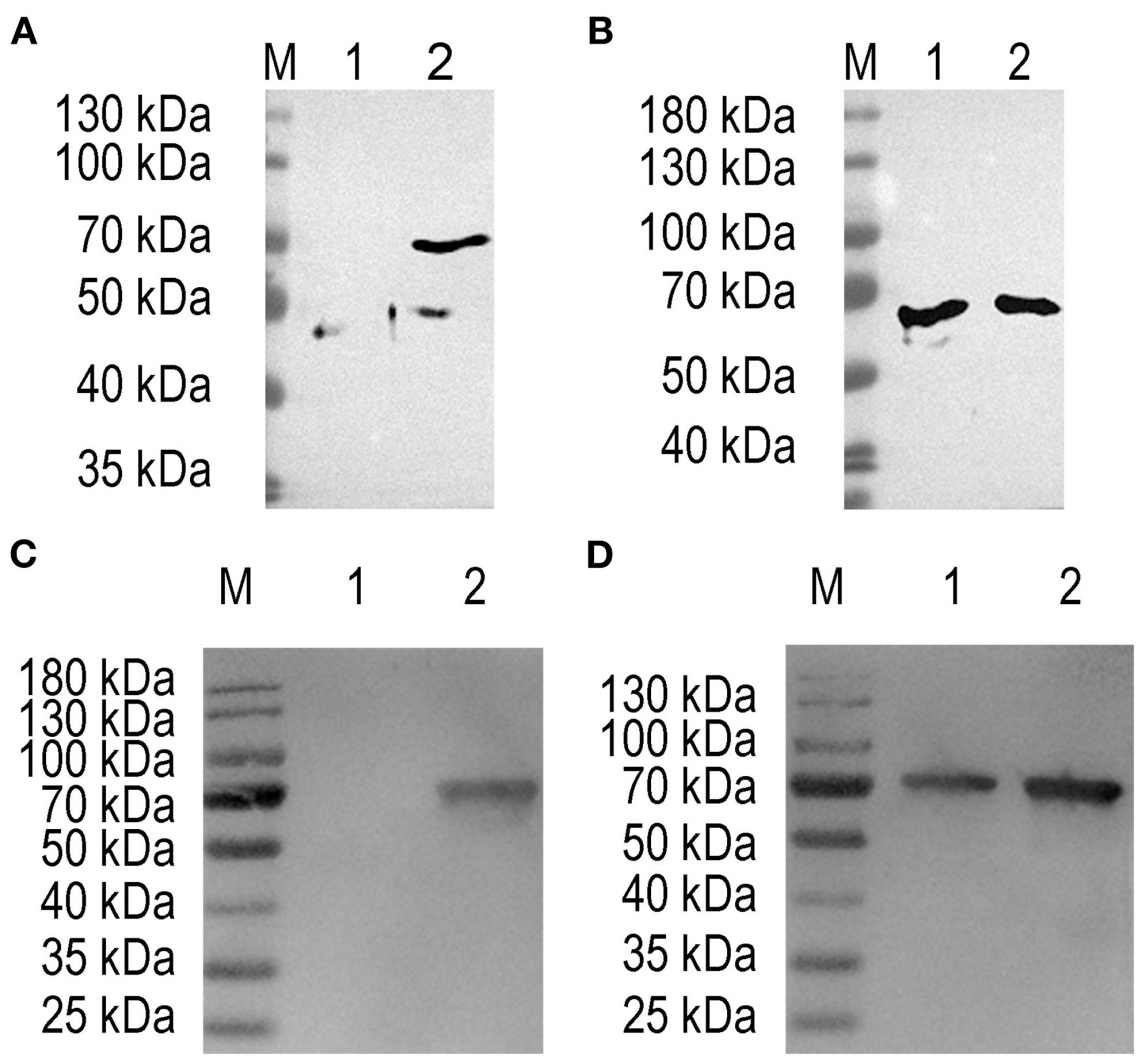
Detection of ΔHexon protein expressed in *Lactococcus lactis* and *Enterococcus faecalis* using western blot. Proteins from ΔHexon-expressing *L. lactis* or ΔHexon-expressing *E. faecalis* was separated by SDS-PAGE, transferred to nitrocellulose membranes, then reacted with polyclonal antisera against ΔHexon protein. **(A)** Detection of ΔHexon protein in NZ9000/pTX8048-SP-ΔHexon-CWA. Lane M, protein molecular weight prestained marker (Thermo Scientific™); Lane 1, ΔHexon protein in NZ9000/pTX8048-SP-ΔHexon-CWA without induction by nisin (Sigma-Aldrich); Lane 2, ΔHexon protein (70 kDa) from NZ9000/pTX8048-SP-ΔHexon-CWA induced by 5 ng/mL nisin (Sigma-Aldrich). **(B)** Detection of ΔHexon protein in MDXEF-1/ΔHexon-CWA. Lane M, Protein molecular weight prestained marker (Thermo Scientific™); Lane 1, ΔHexon protein (70 kDa) in MDXEF-1/ΔHexon-CWA without induction by nisin (Sigma-Aldrich); Lane 2, ΔHexon protein (70 kDa) in MDXEF-1/ΔHexon-CWA induced by 5 ng/mL nisin (Sigma-Aldrich). **(C)** Detection of ΔHexon protein in NZ9000/DC-ΔHexon-CWA. Lane M, protein molecular weight prestained marker (Thermo Scientific™); Lane 1, ΔHexon protein in MDXEF-1/ΔHexon-CWA protein without induction by nisin (Sigma-Aldrich); Lane 2, ΔHexon protein (72 kDa) in NZ9000/DC-ΔHexon-CWA induced by 5 ng/mL nisin (Sigma-Aldrich). **(D)** Detection of ΔHexon protein (72 kDa) in MDXEF-1/DC-ΔHexon-CWA without induction by nisin (Sigma-Aldrich). Lane M, protein molecular weight prestained marker (Thermo Scientific™). Lane 1, ΔHexon protein (72 kDa) in MDXEF-1/DC-ΔHexon-CWA without induction by nisin (Sigma-Aldrich). Lane 2, ΔHexon protein (72 kDa) in MDXEF-1/DC-ΔHexon-CWA induced by 5 ng/mL nisin (Sigma-Aldrich).

**Figure 4:**
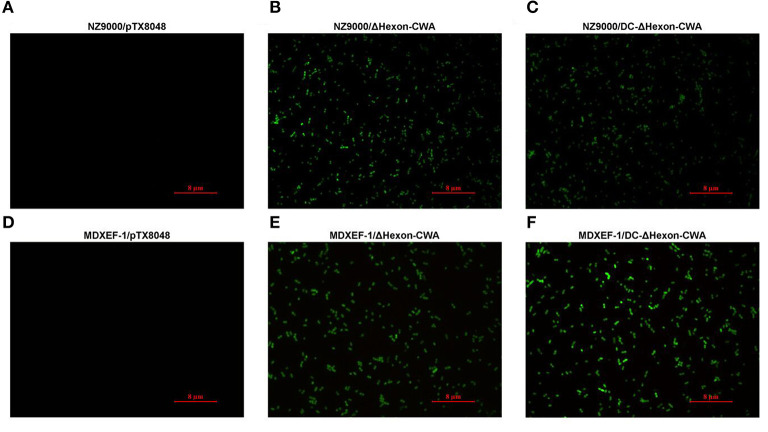
Detection of ΔHexon protein expressed on the surface of recombinant *Lactococcus lactis* NZ9000 and *Enterococcus faecalis* MDXEF-1 by indirect immunofluorescence assay (IFA). The recombinant *L. lactis* and *E. faecalis* induced by 5 ng/mL nisin (Sigma-Aldrich) were washed and centrifuged. The pellets were incubated with rabbit polyclonal antisera against ΔHexon, then reacted with goat anti-rabbit IgG conjugated to fluorescein isothiocyanate (FITC). The fluorescence on the surface of recombinant bacteria NZ9000/ΔHexon-CWA and MDXEF-1/ΔHexon-CWA **(B,E)**, NZ9000/DC-ΔHexon-CWA and MDXEF-1/DC-ΔHexon-CWA **(C,F)** were observed. Recombinant bacteria NZ9000/pTX80488 (31) **(A)** and MDXEF-1/pTX8048 (25) **(D)** were used as negative controls.

The target fragment of 1,230 bp was released from plasmid pTX8048-SP-ΔHexon-CWA and pTX8048-SP-DC-ΔHexon-CWA upon digestion with *Bam*H I and *Kpn* I, respectively. All the positive plasmids were further sequenced. The positive plasmids were electroporated into *L. lactis* NZ9000 and *E. faecalis* MDXEF-1, respectively, and the positive bacteria NZ9000/pTX8048-SP-ΔHexon-CWA, NZ9000/pTX8048-SP-DC-ΔHexon-CWA, MDXEF-1/pTX8048-SP-ΔHexon-CWA, and MDXEF-1/pTX8048-SP-DC-ΔHexon-CWA were identified. The cell wall-anchored ΔHexon protein (70 kDa) fusion or without fusion with DC targeting peptide in *L. lactis* NZ9000 (Figures 3A,C) and *E. faecalis* MDXEF-1 (Figures 3B,D) were detected by western blot. The results from indirect immunofluorescence assay (IFA) showed that fluorescence on the surface of recombinant bacteria NZ9000/ΔHexon-CWA and MDXEF-1/ΔHexon-CWA (Figures 4B,E), NZ9000/DC-ΔHexon-CWA and MDXEF-1/DC-ΔHexon-CWA (Figures 4C,F) were observed.

### Humoral Immune Responses Induced by ΔHexon-Expressing Bacteria

**Figure 5:**
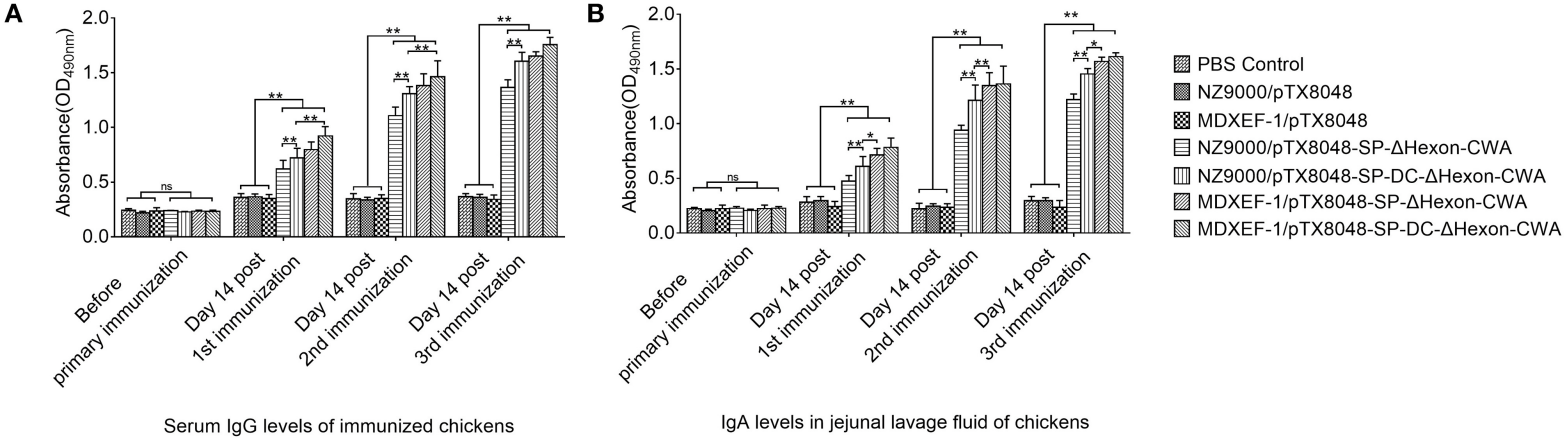
Detection of ΔHexon-specific IgG antibody levels in sera **(A)** and ΔHexon-specific sIgA antibody levels in jejunal lavage fluid **(B)** from chickens immunized with ΔHexon-expressing bacteria in each group. Recombinant ΔHexon protein in *E. coli* BL21 (10 μg/mL) was coated (100 μL per well). On day 14 post each immunization, ELISA method was used to determine the IgG levels in sera **(A)** and sIgA levels in jejunal lavage fluid **(B)**. The values represent mean ± SD (*n* = 5). *p < 0.05, **p < 0.01.

On day 14 post each immunization, the levels of both Hexon-specific sera IgG (Figure 5A) and sIgA in jejunal lavage fluid (Figure 5B) from chickens in the four groups NZ9000/ΔHexon-CWA, NZ9000/DC-ΔHexon-CWA, MDXEF-1/ΔHexon-CWA, and MDXEF-1/DC-ΔHexon-CWA were significantly higher than that in the groups NZ9000/pTX8048, MDXEF-1/pTX8048 and PBS (*p* < 0.01). Notably, the levels of specific IgG and sIgA in the two groups NZ9000/DC-ΔHexon-CWA and MDXEF-1/DC-ΔHexon-CWA were significantly higher than that in the groups NZ9000/ΔHexon-CWA and MDXEF-1/ΔHexon-CWA (*p* < 0.01). The results displayed that introduction of DCpep (DC-ΔHexon-CWA) stimulated more robust humoral immune responses than ΔHexon-CWA.

### mRNA Expression of Cytokines in Spleen

**Figure 6:**
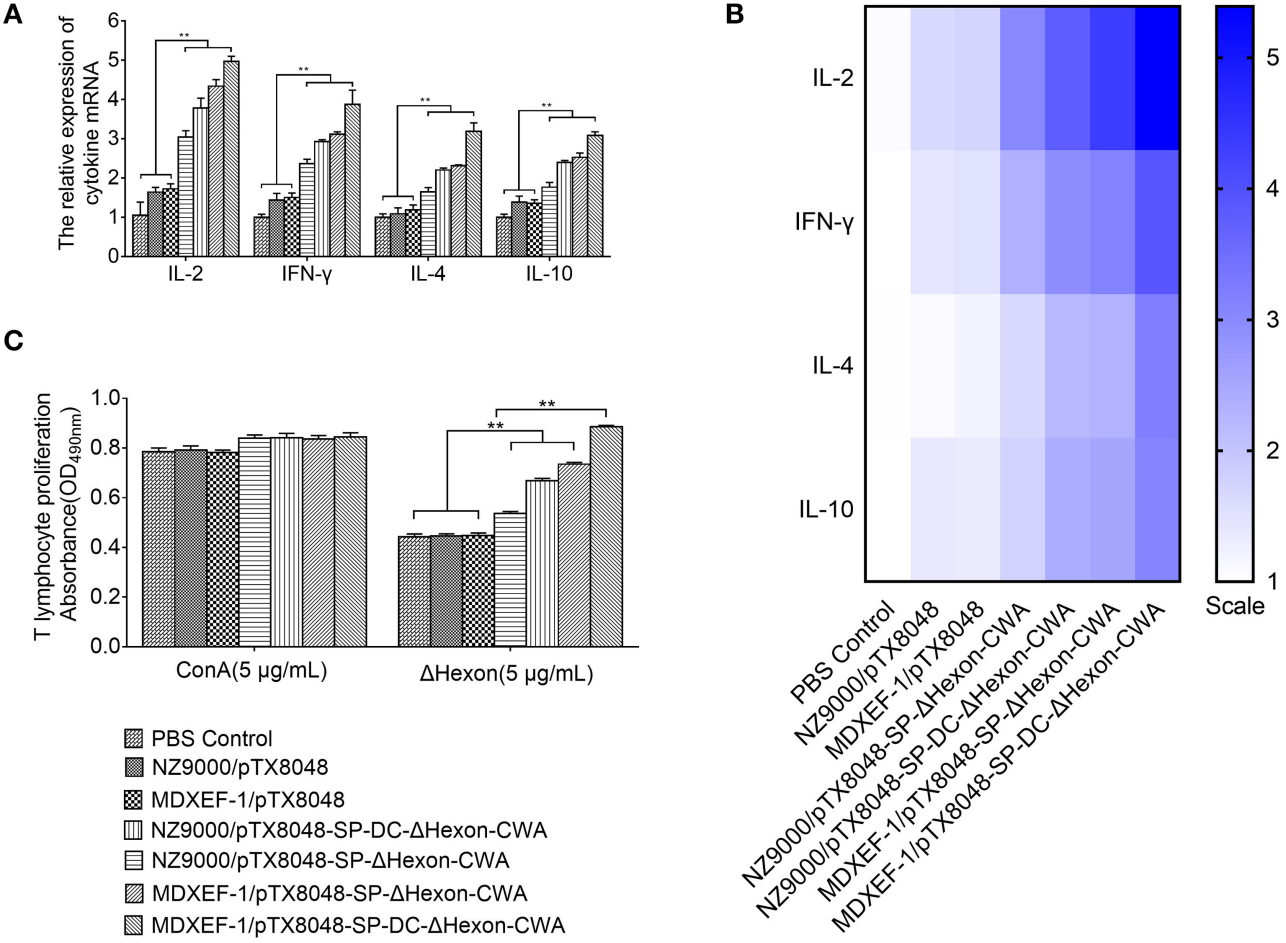
The mRNA expression levels of IL-2, interforn–γ (IFN-γ), IL-4, and IL-10 **(A,B)** in spleens of chickens in each group post-tertiary immunization and the proliferation of PBLs **(C)**. **(A)** On 2-week post-tertiary immunization, the mRNA expression levels of IL-2, interforn-gamma (IFN-γ), IL-4, and IL-10 in spleens of five chickens in each group was determined by quantitative Real-time PCR (qRT-PCR). The mRNA levels of each chicken in each group were divided by mRNA levels of β-actin of the same chicken to normalize IL-2, IFN-γ, IL-4, and IL-10. **(B)** Heat map of the mRNA expression levels of IL-2, IFN-γ, IL-4, and IL-10 in chickens spleens. **(C)** PBLs of chickens (*n* = 5) in each group were isolated on 2-week post the third immunization. The proliferation of lymphocytes stimulated by ΔHexon protein or concavanalin (ConA) was detected by Cell Counting Kit-8 (CCK8) assay. Data are expressed as mean ± SD (*n* = 5). *p < 0.05, **p < 0.01. AST, aspartate transaminase; ALT, alanine transaminase; ALB, albumin; TP, total protein; and LDH, lactate dehydrogenase.

The mRNA levels of ChIL-2, ChIFN-γ, ChIL-4, and ChIL-10 (Figures 6A,B) in spleens of chickens immunized with four ΔHexon-expressing bacteria were significantly higher than of chickens in PBS and empty vector control groups on days 14 post-third immunizations (*p* < 0.01). Moreover, the mRNA levels of ChIL-2, ChIFN-γ, ChIL-4, and ChIL-10 in MDXEF-1/ΔHexon-CWA and MDXEF-1/DC-ΔHexon-CWA group were significantly higher than that in NZ9000/ΔHexon-CWA, and NZ9000/DC-ΔHexon-CWA group, respectively (*p* < 0.01).

### Lymphocytes Proliferation Responses

On days 14 after three immunizations, PBLs from chickens immunized with four ΔHexon-expressing bacteria showed significant specific responses to recombinant ΔHexon protein compared with that from chickens immunized with the NZ9000/pTX8048, MDXEF-1/pTX8048, and PBS (pH7.2) (*p* < 0.01). However, proliferation responses of PBLs to ConA between any other two groups showed no statistical difference (*p* >0.05; Figure 6C). Proliferation responses of PBLs to ΔHexon protein in MDXEF-1/DC-ΔHexon-CWA and NZ9000/DC-ΔHexon-CWA group were higher than that in MDXEF-1/ΔHexon-CWA and NZ9000/ΔHexon-CWA group, respectively (*p* < 0.01). Notably, proliferation responses of PBLs to ΔHexon protein in the two groups immunized with ΔHexon-expressing *E. faecalis* were higher than those in the two groups immunized with ΔHexon-expressing *L. lactis* (*p* < 0.01; Figure 6C).

### Function Indexes and Virus Load in Liver

**Figure 7:**
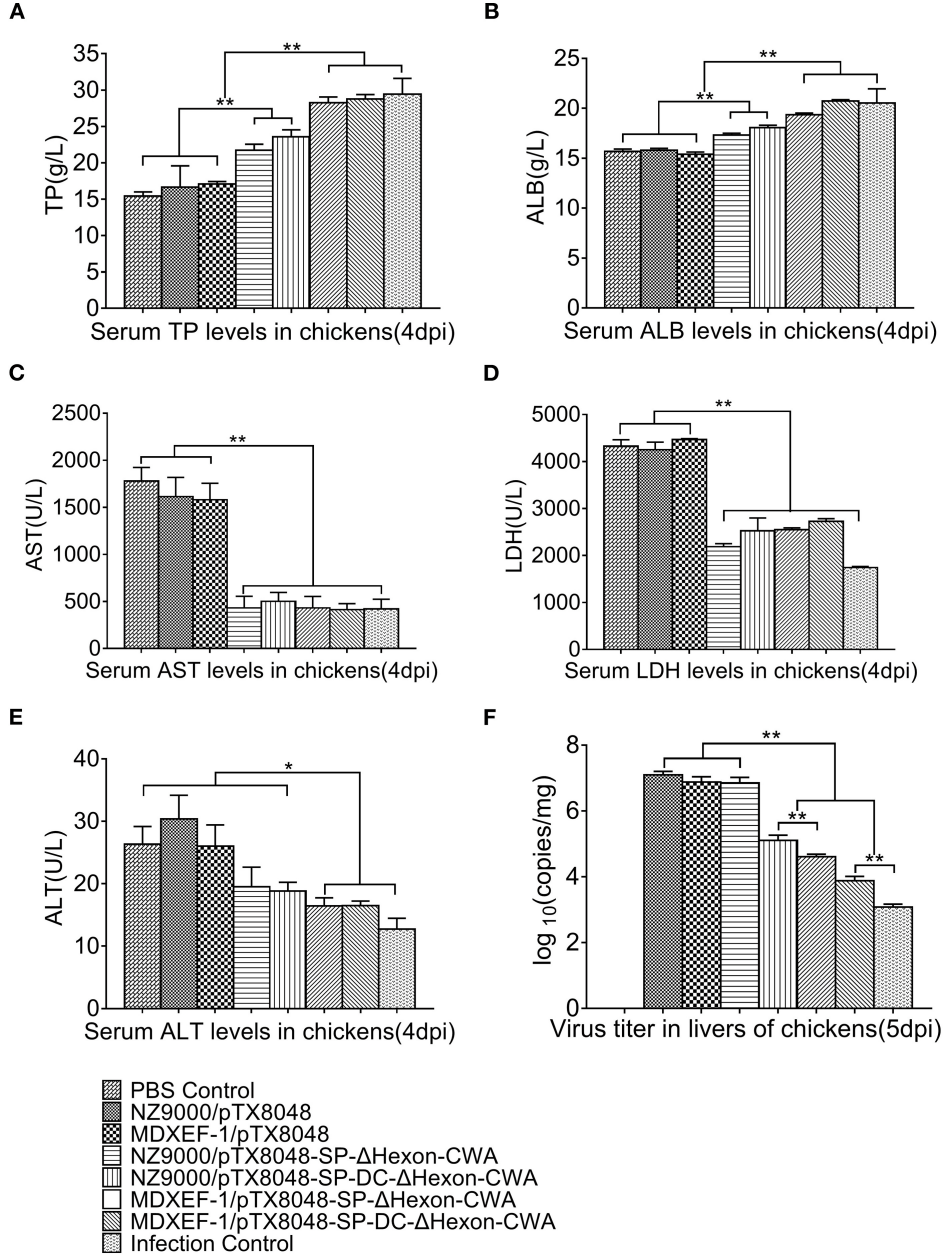
Levels of total protein (TP) **(A)**, albumin (ALB) **(B)**, aspartate transaminase (AST) **(C)**, lactate dehydrogenase (LDH) **(D)**, alanine transaminase (ALT) **(E)** in sera, and copies of FAdV **(F)** in livers of chickens challenged with FAdV-4/GX01. On 4 dpi, the levels of TP **(A)**, ALB **(B)**, AST **(C)**, LDH **(D)**, ALT **(E)** in sera of chickens from each group were detected using a Detection Kit (Jiancheng Bioengineering Institute, Nanjing, China) according to the manufacturer's protocol. **(F)** On 5 dpi, liver samples from chickens in each group were collected. Total DNA was extracted using TaKaRa MiniBEST Viral RNA/DNA Extraction Kit Ver.5.0 (Takara, Beijing, China). qRT-PCR was applied to detect copies of FAdV DNA in samples. Each value represents mean ± SD (*n* = 5). *p < 0.05, **p < 0.01.

Previous studies have shown that the liver function indexes were related to seral TP, ALB, AST, ALT, and LDH, and changed obviously on day 4 post-infection (dpi) (38). On 4 dpi, the levels of TP, ALB, AST, LDH, and ALT (Figure 7) in sera of chickens immunized with four ΔHexon-expressing bacteria showed no statistical differences with chickens in the PBS group. In contrast, significant differences between PBS control group and challenge control group or empty vector control groups (NZ9000/pTX8048 and MDXEF-1/pTX8048) were observed. Specifically, ALB and TP levels in sera of chickens from both challenge control and vector control groups were significantly lower than those in PBS group (*p* < 0.01), while the levels of AST, ALT, and LDH were significantly higher. On 5 dpi, the copies of FAdV in livers of chickens in challenged control group (1.2 × 10^8^ copies/mg) and vector control groups (7.6 × 10^7^ copies/mg for NZ9000/pTX8048, 7.0 × 10^7^ copies/mg for MDXEF-1/pTX8048) were significantly higher than that of chickens immunized with the four ΔHexon-expressing bacteria (1.3 × 10^6^ copies/mg for NZ9000/ΔHexon-CWA, 4.1 × 10^5^ copies/mg for NZ9000/DC-ΔHexon-CWA, 7.6 × 10^4^ copies/mg for MDXEF-1/ΔHexon-CWA and 1.2 × 10^4^ copies/mg for MDXEF-1/DC-ΔHexon-CWA) (*p* < 0.01). Meanwhile, significant differences were observed between groups NZ9000/ΔHexon-CWA and NZ9000/DC-ΔHexon-CWA, and between MDXEF-1/ΔHexon-CWA and MDXEF-1/DC-ΔHexon-CWA (Figure 7F).

### Mortality

**Figure 8:**
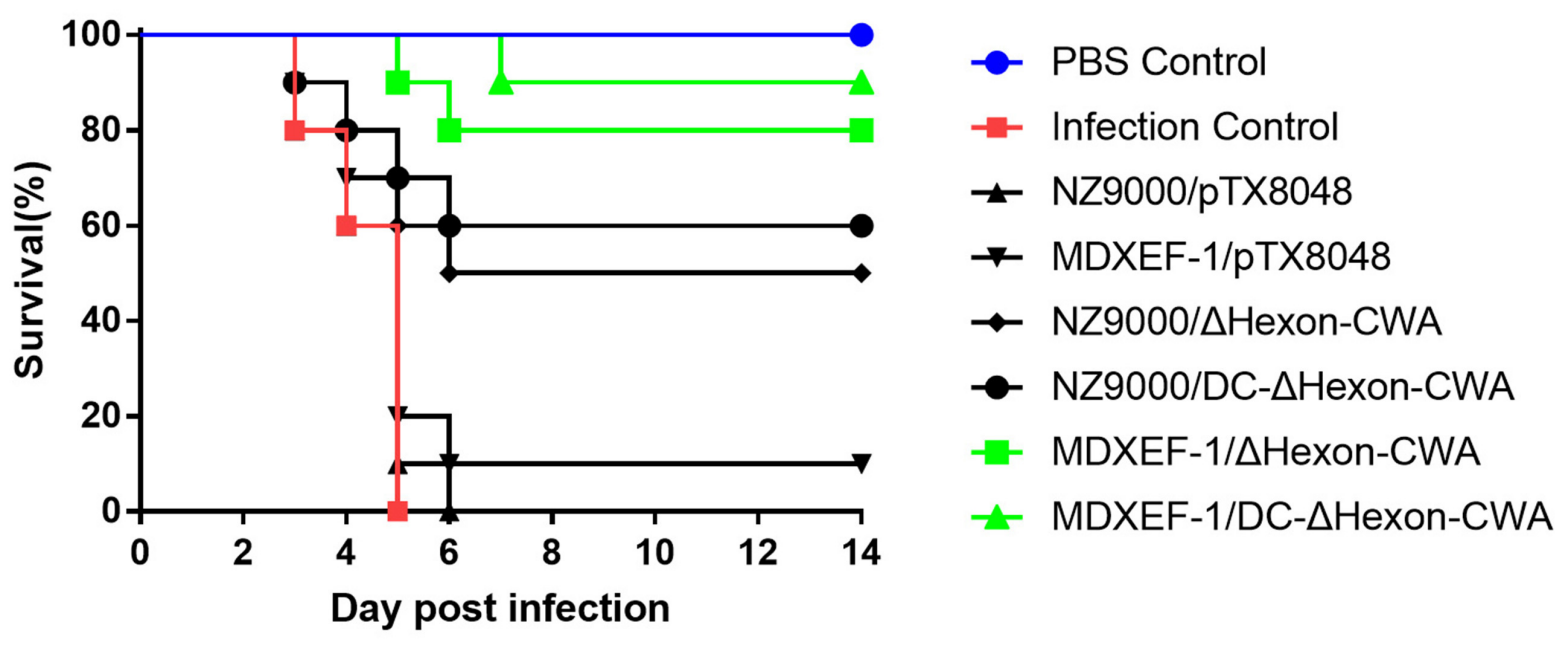
Survival ratios of chickens to challenge of virulent FAdV-4/GX01 in each immunized group. After challenging with virulent FAdV-4/GX01, 90–100% of chickens in the groups were immunized with NZ9000/pTX8048, MDXEF-1/pTX8048, and PBS died. The survival ratio of 50, 60, 80, 90% were observed in chickens immunized with NZ9000/ΔHexon-CWA, NZ9000/DC-ΔHexon-CWA, MDXEF-1/ΔHexon-CWA, and MDXEF-1/DC-ΔHexon-CWA, respectively.

On 3 dpi, chickens in the challenged control group and vector control groups (NZ9000/pTX8048, MDXEF-1/pTX8048) were depressed and gradually dead showing 100% mortality on 5 dpi. Birds in the four groups immunized with ΔHexon-expressing bacteria were partially protected against FAdV challenge in 100% lethal dose (Figure 8), displaying the survival rates of 50, 60, 80, 90% for the group NZ9000/ΔHexon-CWA, NZ9000/DC-ΔHexon-CWA, MDXEF-1/ΔHexon-CWA, and MDXEF-1/DC-ΔHexon-CWA, respectively. Notably, the time of death for challenged chickens in the group MDXEF-1/ΔHexon-CWA and MDXEF-1/DC-ΔHexon-CWA was obviously delayed compared to other immunized groups, indicating live recombinant bacteria MDXEF-1/ΔHexon-CWA and MDXEF-1/DC-ΔHexon-CWA exhibited more protective efficacy against FAdV challenge than the live recombinant bacteria NZ9000/ΔHexon-CWA and NZ9000/DC-ΔHexon-CWA.

### Pathological Lesions

**Figure 9:**
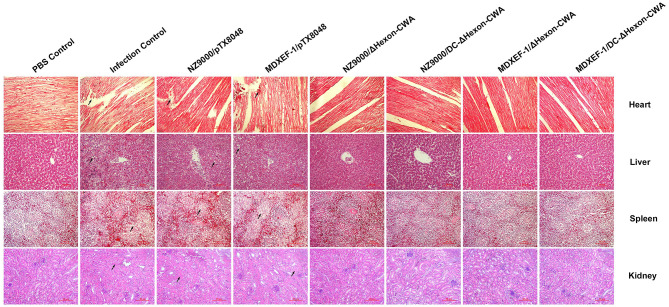
Histopathological changes in heart, liver, spleen, and kidney tissues from chickens at 5 dpi. The prepared slides were stained with hematoxylin and eosin (HE). The typical histopathological changes were recorded in the heart, liver, spleen, and kidney tissues of chickens in the challenged control group and empty vector control groups (NZ9000/pTX8048, and MDXEF-1/pTX8048). The hepatic cells displayed vacuolar degeneration and necrosis, and basophilic inclusion bodies were observed in hepatic cells. Myocardial fibers in heart tissues were rupture and necrosis. Renal tubular epithelial cells in kidney tissues were swelling and degenerating. Lymphocytes in the spleen tissues were severe necrosis. The relative slight histopathological changes in heart, liver, spleen, and kidney tissues were present in the groups immunized with ΔHexon-expressing bacteria (NZ9000/ΔHexon-CWA, NZ9000/DC-ΔHexon-CWA, MDXEF-1/ΔHexon-CWA, and MDXEF-1/DC-ΔHexon-CWA). The hearts, livers, spleens, and kidneys tissues of chickens from the PBS control group showed no histopathological changes. Scale bar = 80 μm. Solid arrows point to lesion areas.

The typical gross lesions of HPS were observed after challenging with a lethal dose of FAdV-4/GX01, showing by typical gross lesions, including hydropericardium, splenomegaly, hepatomegaly, and nephritis during autopsy of challenged chickens in each Group (Supplementary Figure 1). Of note, chickens from the four groups immunized with live recombinant ΔHexon-expressing bacteria presented mild HPS, and the gross pathological lesions in hearts, spleens, livers, and renals were also moderate. The typical histopathological changes of HPS were presented by the myocardial fiber fracture and even dissolution, lymphocytic myocarditis, degeneration and even necrosis of renal tubular epithelium, and severe necrosis disintegration of splenic lymphocytes. However, chickens from the four groups immunized with ΔHexon-expressing bacteria, especially the group vaccinated with MDXEF-1/DC-ΔHexon-CWA, displayed mild histopathological changes (Figure 9).

## Discussion

The structural proteins of FAdV-4 contain Fiber 1, Fiber 2, Penton, and Hexon (39, 40). The subunit vaccines based on Penton and Fiber 2 were recorded to induce immunoprotection against FAdV infection in chickens (22). However, the immune protection and immune mechanism of the vaccine based on Hexon protein was not reported until now. Hexon protein, one of the most important structural proteins on the surface of adenovirus, contains the type-, group- and subclass-specific antigenic determinants that can stimulate the body to produce efficient neutralizing antibodies. In the present study, the truncated Hexon protein (ΔHexon) of the current epidemic FAdV-4 strain (isolated and stored in our lab) spanning amino acids from 10 to 420 was chosen, in which loop1, loop2, and P1 were contained. Prediction using molecular software DNAstar Lasergene 7.1 (DNAS Inc., Madison, WI, USA) showed that ΔHexon displays high antigenicity. Meanwhile, ΔHexon protein was fused to dentritic cell (DC) targeting peptide (DC-ΔHexon) (21, 41) with the aim of enhancing ΔHexon-specific immune responses. Then fusion protein DC-ΔHexon was inserted into expressing vector (31) in which cell wall-anchored (CWA) sequences were contained to display target fusion protein on the surface of recombinant *L. lactis* and *E. faecalis* (25, 31).

The results of vaccinations suggested that all four recombinant ΔHexon-expressing bacteria elicited a higher level of sera IgG and sIgA in jejunal lavage compared to the empty control group (*p* < 0.01), demonstrating that humoral immune responses of immunized chicken were effectively evoked. Moreover, cell wall-anchored ΔHexon protein fused by DC targeting peptide (DCpep) induced higher sIgA levels in jejunal lavage than cell wall-anchored ΔHexon delivered by *L. lactis* and *E. faecalis* (*p* < 0.05), which indicating that introduction of DCpep on the surface of bacteria effectively targeted intestinal dendritic cells to enhance antigenic uptake and the subsequent delivery to immune cells. The above results were consistent with our previous report by Ma et al. (31). In addition, live recombinant bacteria MDXEF-1/ΔHexon-CWA and MDXEF-1/DC-ΔHexon-CWA induced more robust immune responses and provided more protective effects than NZ9000/ΔHexon-CWA and NZ9000/DC-ΔHexon-CWA, respectively (*p* < 0.05). The possible explanations for this result may be that *E. faecalis* was isolated from ceca and partially colonized in ceca (data not published), and live recombinant ΔHexon-expressing sustainably stimulated ΔHexon-specific immune responses. Meanwhile, ΔHexon-CWA, or DC-ΔHexon-CWA protein in recombinant ΔHexon-expressing *E. faecalis* could be detected without induction of nisin, which suggests that host bacteria *E. faecalis* MDXEF-1 probably produce nisin or nisin-like substance that continuously induced expression of the target protein. This prediction remains to be further studied and proved in our subsequent research. Previous study has demonstrated that adenovirus settles in the intestinal epithelium at 12 h post-infection *via* oral route, and the virus can be detected in blood as early as 24 h post-infection (42). The significantly higher levels of Hexon-specific IgG could bind to FAdV in sera to form complexes that could be more easily engulfed by macrophages. The higher levels of sIgA in jejunal lavage can bind to FAdV located at local intestinal epithelium and intercept virus invasion upon initial infection. Therefore, chickens vaccinated with the four recombinant bacteria expressing anchored ΔHexon protein, especially those carrying DCpep bacteria, induced more effective systemic humoral immune responses and protection against FAdV infection.

As for cellular immune responses, in the present study, the proliferation response of PBLs was more efficient in the groups immunized with four ΔHexon-expressing bacteria. Moreover, mRNA levels of ChIL-2, ChIFN-γ, and ChIL-4, ChIL-10 in spleens of chickens immunized with four ΔHexon-expressing bacteria were significantly upregulated, suggesting that both the Th1 and Th2 type responses were enhanced to resist virus infection. The present results could support the above analysis that on day 5 post-infection (dpi), the number of FAdV copies in livers of chickens from NZ9000/pTX8048 and MDXEF-1/pTX8048 group was significantly higher than that in the four ΔHexon-expressing bacteria groups, especially the two groups of ΔHexon-expressing *E. faecalis*.

The average AST, ALT, and LDH levels in sera of chickens in NZ9000/pTX8048, MDXEF-1/pTX8048 group, and infection control group were transiently and significantly elevated on 4 dpi compared to PBS control group and four ΔHexon-expressing bacteria groups, which indicating that severe pathological changes have occurred in liver (43). Meanwhile, the significant decrease in the levels of TP and ALB also suggested that protein synthesis was affected, which further demonstrated that the liver had been seriously damaged. This result is consistent with the previous research (38). Furthermore, the little-changed indexes of liver function in the four groups of chickens immunized with ΔHexon-expressing bacteria also proved the protective effects against FAdV infection.

In the present study, on 5 dpi, typical pathological and histopathological changes were accordingly observed in the hearts, livers, and kidneys of chickens in the challenged control group, NZ9000/pTX8048 and MDXEF-1/pTX8048 group, including hydropericardium, splenomegaly, hepatomegaly, and nephritis, all of which are also recorded in other reports (43, 44). The pathological changes in hearts, livers, and kidneys of chickens in the four groups immunized with ΔHexon-expressing bacteria were relatively slight, which suggested that the live bacteria expressing ΔHexon-CWA protein protected against pathological injury of FAdV targeted organs. However, a 100% protection ratio was not observed in the present study, which may be explained by the fact that the virus strain FAdV-4/GX01 is highly virulent, and chickens were challenged by intramuscular injection way. In our preliminary test, chickens challenged with the lethal dose of FAdV-4/GX01 *via* intramuscular injection caused mortality of 90–100%, which is close to the reported highest clinical mortality rate (45). The present results showed that the constructed four recombinant bacteria, especially *E. faecalis* expressing ΔHexon protein, provided promising protection against the FAdV-4 infection.

Besides, it is worth noting that the viral load in the livers of the challenged chickens in the four groups immunizing with ΔHexon-expressing bacteria is still high, although it is significantly lower than that in the infection control group. Recently, several studies have shown that Fiber 2 plays a vital role during the invasion of FAdV into host cells (46, 47). In some reports, the co-immunization of recombinant LAB expressing several target proteins is more promising (48, 49). Currently, another four kinds of live recombinant LAB expressing Fiber 2 protein have been constructed in our lab, and the subsequent co-immunization of live recombinant *E. faecalis* expressing ΔHexon and Fiber 2 would be further explored.

Overall, these results demonstrated that recombinant *L. lactis* and *E. faecalis* delivering ΔHexon protein could evoke strong systemic immune responses against FAdV infection, relieve pathological injury and functional damage in target organs, reduce virus load in liver to some extent, and prevent HPS caused by FAdV infection. The data presented in this report provide references for exploring potential vaccines for HPS.

## Data Availability Statement

All data generated for this study are original and included in this article and its Supplementary Material. Any further inquiries may be directed to the corresponding author Dexing Ma.

## Ethics Statement

The protocol of animal experiments was approved by the Animal Ethics Committee of Northeast Agricultural University NEAU-2018-09-0232-12.

## Author Contributions

DM and ZJ designed the study. GL, CM, XP, XY, and ZJ prepared experimental materials. CM contributed to analytic tools. ZJ and CM analyzed the data and wrote the paper. ZJ, DM, CM, and GL revised the manuscript. All authors reviewed the results and approved the final version of the manuscript.

## Conflict of Interest

The authors declare that the research was conducted in the absence of any commercial or financial relationships that could be construed as a potential conflict of interest.
