# Supplementary material for: Oral Immunization of Recombinant Lactococcus lactis and Enterococcus faecalis Expressing Dendritic Cell Targeting Peptide and Hexon Protein of Fowl Adenovirus 4 Induces Protective Immunity Against Homologous Infection
Source: Front Vet Sci. 2021 Feb 23;8:632218. doi: 10.3389/fvets.2021.632218 (PMC7940690; doi:10.3389/fvets.2021.632218)
Supplement: Supplementary file 1 [file Data_Sheet_1.docx]

Supplementary Material

# Supplementary Figures and Tables

## Supplementary Figures


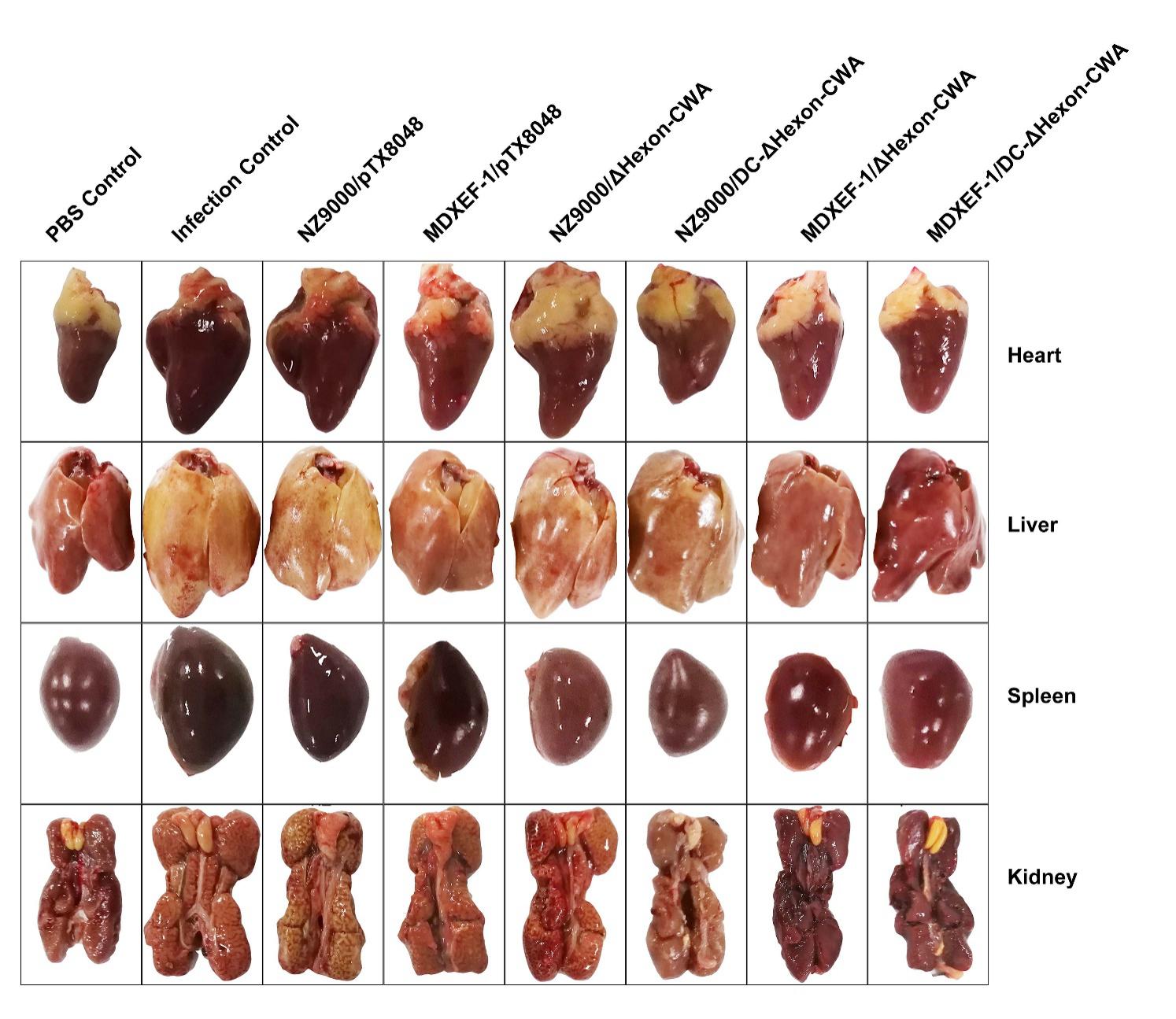


**Supplementary Figure 1.** Gross pathological lesions in hearts, livers, spleens, and kidneys from chickens in each group. The noticeable gross pathological lesions in hearts, livers, spleens, and kidneys of chickens from the challenged control group and empty control group (NZ9000/pTX8048 and MDXEF-1/pTX8048) were observed, including hemorrhage in epicardium and hydropericardium, swollen and friable livers with multifocal areas of necrosis, and mild or severe enlargement in the spleens and kidneys. The gross pathological lesions in hearts, livers, spleens, and kidneys of chickens from the groups immunized with ΔHexon-expressing bacteria (NZ9000/ΔHexon-CWA, NZ9000/DC-ΔHexon-CWA, MDXEF-1/ΔHexon-CWA, and MDXEF-1/DC-ΔHexon-CWA) were relatively mild.

## Supplementary Tables

Supplementary Table 1 Experimental design of immunizations and challenge.

|  |  | **Immunization** | | | | | **Challenge** |
| --- | --- | --- | --- | --- | --- | --- | --- |
| **Groups** | **Number** | **Primary** | | **Secondary** | **Third** | | **Challenge at** |
|  |  | **day7, 8 and 9** | **day21, 22 and 23** | | | **day35, 36 and 37** | **days 49** |
| PBS Control | 40 | PBS (pH7.2) | | | | | PBS (pH7.2) |
| NZ9000/pTX8048 | 30 | *L. lactis* NZ9000/pTX8048, 1× 10^10^CFU | | | | | 10^5.2^ ELD_50_ |
| MDXEF-1/pTX8048 | 30 | *E. faecalis* MDXEF-1/pTX8048, 5× 10^9^CFU | | | | | 10^5.2^ ELD_50_ |
| NZ9000/ΔHexon-CWA | 30 | *L. lactis* NZ9000/pTX8048-SP-ΔHexon-CWA, 1× 10^10^CFU | | | | | 10^5.2^ ELD_50_ |
| NZ9000/DC-ΔHexon-CWA | 30 | *L. lactis* NZ9000/pTX8048-SP-DC-ΔHexon-CWA, 1× 10^10^CFU | | | | | 10^5.2^ ELD_50_ |
| MDXEF-1/ΔHexon-CWA | 30 | *E. faecalis* MDXEF-1/pTX8048-SP-ΔHexon-CWA, 5× 10^9^CFU | | | | | 10^5.2^ ELD_50_ |
| MDXEF-1/DC-ΔHexon-CWA | 30 | *E. faecalis* MDXEF-1/pTX8048-SP-DC-ΔHexon-CWA, 5× 10^9^CFU | | | | | 10^5.2^ ELD_50_ |
| Infection Control | 10 | PBS (pH7.2) | | | | | 10^5.2^ ELD_50_ |
